# Gastrointestinal cell injury and perceived symptoms after running the Boston Marathon

**DOI:** 10.3389/fphys.2023.1268306

**Published:** 2023-10-16

**Authors:** Melani R. Kelly, Dawn M. Emerson, Brendon P. McDermott, Whitley C. Atkins, Cory L. Butts, R. Mark Laursen, Christopher Troyanos, Andrew Duckett, Jacob Siedlik

**Affiliations:** ^1^ Department of Health, Sport, and Exercise Sciences, University of Kansas, Lawrence, KS, United States; ^2^ Department of Exercise Science and Outdoor Recreation, Utah Valley University, Orem, UT, United States; ^3^ Department of Exercise Science, University of South Carolina, Columbia, SC, United States; ^4^ Department of Health, Human Performance and Recreation, University of Arkansas, Fayetteville, AR, United States; ^5^ Department of Exercise and Nutrition Sciences, Weber State University, Ogden, UT, United States; ^6^ Sargent College of Health and Rehabilitation Sciences, Boston University, Boston, MA, United States; ^7^ Boston Athletic Association, Boston, MA, United States; ^8^ Department of Athletics, Boston University, Boston, MA, United States; ^9^ Department of Exercise Science and Pre-Health Professions, Creighton University, Omaha, NE, United States

**Keywords:** endurance exercise, intestinal fatty acid binding protein, hydration, race medicine, intestinal epithelium

## Abstract

Gastrointestinal (GI) disturbances are a prevalent cause of marathon related complaints, and in extreme cases can promote life-threatening conditions such as exertional heat stroke. Our aim was to study intestinal cell injury [via intestinal fatty acid binding protein (I-FABP)] and perceived GI distress symptoms among marathon runners. We also examined potential risk factors (e.g., inadequate sleep) that could exacerbate GI disturbances in healthy, trained endurance runners. This was a parallel mixed-methods study design. 2019 Boston Marathon participants were recruited via email and subjects completed surveys before the race describing demographics and training history. Participants completed a GI questionnaire to assess presence and severity of symptoms, a survey regarding risk factors (e.g., recent illness, medications) that could promote GI disturbances, and provided a urine sample at three time points (immediately pre-race, post-race, and 24-h post-race). Due to weather, blood samples were only collected immediately and 24-h post-race. A total of 40 runners (males: *n* = 19, age = 44.9 ± 10.8 years; females: n = 21, age = 44.8 ± 10.6 years) completed this study. I-FABP significantly decreased from post-race (3367.5 ± 2633.5 pg/mL) to 24-h post-race (1657.3 ± 950.7 pg/mL, t (39) = −4.228, *p* < .001, d = −.669). There was a significant difference in overall GI symptom scores across the three time points (F (2, 39) = 41.37, *p* < .001). The highest average score occurred post-race (.84 ± .68), compared to pre-race (.09 ± .12) and 24-h post-race (.44 ± .28). Post-race I-FABP (r = .31, *p* = .048) and post-race urine specific gravity (r = .33, *p* = .041) were significantly correlated with post-race GI symptom scores. Our study provides further support to the individualized nature of GI disturbances, with participants experiencing a wide range of risk factors that can influence the extent of GI damage and perceived symptoms during and after exercise.

## Introduction

With the increasing popularity of marathon running across the world, extensive work has been done to limit factors known to decrease a runners’ performance or predispose them to a medical event during exercise. Despite advanced preparation and precautions, gastrointestinal (GI) distress (e.g., abdominal pain, nausea) continues to be an issue, often threatening a runner’s ability to continue exercise (Keeffe et al., 1984; Hölmich et al., 1988; Riddoch and Trinick, 1988; Halvorsen et al., 1990; Pugh et al., 2018). Although there are several mechanisms to induce GI distress, two primary concerns involve reduced splanchnic blood flow that causes GI ischemia and loss of the GI epithelial barrier integrity (Van Wijck et al., 2012a).

Some studies report GI blood flow reduces up to ∼80% during exercise (Rowell, 1974), and blood flow occlusion linearly increases with greater exercise intensity, exercise duration and/or environmental heat strain (Rowell, 1974; March et al., 2017). When the GI tract is in an ischemic state, tissues are directly damaged, and an inflammatory response is initiated to repair tissues. Evidence also suggests that when blood flow is reestablished–reperfusion–cells continue to experience necrosis and a cascade of inflammatory mediators (e.g., pro-inflammatory cytokines, neutrophils, adhesion molecules) are signaled to repair damaged tissue (Carden and Granger, 2000). Either through direct cell damage or secondary damage from inflammatory mediators, ischemia-reperfusion damages GI tissues, including those responsible for maintaining GI barrier integrity (van Wijck et al., 2011a).

The GI epithelial barrier is maintained by tight junctions, adherens junctions, and desmosomes–collectively known as the apical junctional complex (Balda et al., 1992; Yu and Yang, 2009). Tight junctions are the key regulators for paracellular permeability, which prevents larger molecules, like endotoxin (i.e., lipopolysaccharide [LPS]), from leaving the GI tract (Lambert, 2009; Turner, 2009). When the epithelial barrier is functioning appropriately, LPS is contained within the GI tract, with only small amounts entering systemic circulation. In healthy individuals, or when exercise stressors are manageable, the immune system and liver effectively clear LPS from the blood. However, when the GI barrier is damaged enough, higher concentrations of LPS are allowed into the blood, overwhelming the ability for the liver and immune system to filter LPS out. As a result, LPS builds up systemically, promoting core temperature increases and potentially leading to a medical emergency referred to as endotoxemia (Lambert, 2008; Lambert, 2009).

The prevalence of GI distress symptoms varies considerably between individuals and many factors (e.g., poor sleep, inappropriate nutrition, hypohydration) may exacerbate symptom severity. For example, exercising in a hypohydrated state further reduces splanchnic blood flow, exacerbating ischemia related damage (Costa et al., 2019). An individual exercising after a recent illness will have an active immune response, which may inhibit their ability to maintain epithelial barrier integrity and neutralize LPS (Lim and Mackinnon, 2006; Selkirk et al., 2008). Food intake can also elicit GI distress. High carbohydrate or fat intake immediately prior to or during a race is associated with GI discomfort due to altered digestion rates (Rehrer et al., 1992; Shi et al., 2004; Etxebarria et al., 2021). GI symptoms may also be reported due to other factors such as decreased immune function (Nieman et al., 1990) and physical fitness level (Sakurada and Hales, 1998; Murray, 2006; Jeukendrup and McLaughlin, 2011).

One major limitation to understanding how and when different factors affect an individual’s exercise-induced GI distress is a lack of pre-race screening and medical data from races (Nieman et al., 1990; Gardner et al., 1996; Cleary, 2007; Patel et al., 2016; Stearns et al., 2020). Without pre-event information, we cannot determine whether a recent illness, pre-existing medical condition, poor sleep, or medication use may be associated with the runner’s GI distress. Feasibility limits road race coordinators ability to perform pre-race medical screening, meaning medical personnel have little knowledge about the multiple intrinsic risk factors runners may enter a race with that predispose them to medical issues during the event (Schwellnus and Derman, 2014). It may not be practical for some medical personnel or race coordinators to collect pre-race information to gain a better understanding of some of the intrinsic factors or underlying medical conditions runners enter a race with, and sometimes intra-race strategies are to blame for an individual needing medical attention. Regardless, by examining different risk factors, we can potentially reduce the occurrence and severity of GI distress among runners, as well as decrease the burden on medical personnel tasked with assisting these individuals.

As previously mentioned, not only is the body actively repairing damaged tissues caused by exercise, the blood flow redistribution back to the GI tract after elicits damage and an inflammatory response (Hill et al., 2020). For this reason, a runner’s health and performance can be negatively impacted for hours or days after exercise. Researchers and clinicians must try to understand exercise-induced GI distress beyond the exercise bout by exploring how individuals respond and recover in the hours after activity. The types of activities and behaviors runners engage in after exercise are of particular interest, as they may directly relate to the ability to regain GI barrier integrity and/or decrease the prevalence and incidence of exercise-induced distress symptoms. For instance, an individual experiencing GI symptoms (e.g., nausea, abdominal pain) may also intentionally restrict food or fluid intake in fear of adding to their symptomology, further delaying the recovery process and promoting hypohydration and inadequate energy availability. Or, if after the race, the runner received less than the recommended 7 h of sleep (CDC), epithelial barrier recovery may be delayed and distress symptoms remain the day after.

The continued popularity of running events and high prevalence of GI disturbance reported among marathon runners (Keeffe et al., 1984; Hölmich et al., 1988; Riddoch and Trinick, 1988; Halvorsen et al., 1990; Pugh et al., 2018) requires evidence-based recommendations to limit GI distress and better understand how GI damage and GI symptoms are influenced by individual risk factors. Not only does limiting the extent of a runner’s GI distress improve performance and overall race experience, the link between intestinal cell injury and more life-threatening conditions, such as endotoxemia and exertional heat stroke (EHS), means that limiting GI damage could also limit the risk for these serious medical conditions. The overall purpose of our study was to begin exploring different risk factors associated with GI damage among marathon runners and how these factors influenced GI distress during and after the race. We had three specific aims: 1) examine the changes in intestinal cell injury on race day and the day after the race, 2) examine the incidence and severity of perceived GI distress symptoms on race day and the day after the race, and 3) examine whether risk factors (e.g., sleep quantity, food intake, recent illness) were associated with intestinal cell injury and GI distress symptoms on race day and the day after the race.

## Materials and methods

This was a parallel mixed-methods study design.

## Participants

Registered 2019 Boston Marathon runners were invited to participate in this study via an email sent by Boston Athletic Association race organizers. All registered runners received the email about 1 month before the race. Interested runners contacted a primary investigator and were sent a health history screening questionnaire (HHQ) to determine eligibility. Inclusion criteria required participants have no previous vasovagal response during blood draws, be between the ages of 18–65 years old, and have either qualified to run the Boston Marathon based on age or ran a marathon in the past 12 months under 4 h. Individuals were excluded if they had any current cardiovascular, respiratory, GI, bleeding, inflammatory, metabolic, or fluid-electrolyte disorder or other chronic disease. Individuals using selective cyclooxygenase-2 inhibitors, anti-hypertensives, or other medications that affect kidney, GI, or cardiovascular function, or fluid-electrolyte balance were excluded. Participants were also excluded if they would not be able to attend a 24-h post-race data collection session. Elite runners and non-qualifying runners (e.g., charity runner) were not included in the study. This study was approved by the primary investigators’ Institutional Review Boards and participants consented prior to participation.

### Instruments and protocols

#### Participant screening

We used a 65-question HHQ to screen potential participants. The HHQ was delivered through an online platform (Qualtrics, Provo, UT). The prevalence of pre-existing medical conditions was determined by using the Physical Activity Readiness Questionnaire for Everyone (PAR-Q). The PAR-Q is a 28-question self-administered pre-participation risk screening tool to assess physical activity and exercise participation (You, 2002). If a participant answered “yes” to any of the questions, they were asked to follow up with an explanation or list the specific medical condition in a manual entry text box.

We added additional questions to the HHQ to further determine eligibility to participate and characterize medical history. Three questions asked whether the individual experienced a previous exertional heat illness (EHI). If yes, the individual was asked type of EHI (exertional heat exhaustion and/or EHS) and date of most recent occurrence. Three questions assessed if participants had a current illness (e.g., sinusitis, influenza) and/or current symptoms (e.g., fever, headache) while taking the survey. Eleven questions assessed medication and supplement use, including type and dose of prescribed and over-the-counter (OTC) medications and types of supplements and/or vitamins currently used. Twelve demographic questions identified age, self-reported height and weight, where they lived, race, and sex. Females were asked four additional questions regarding menstrual cycle regularity. Finally, five additional questions assessed how participants entered the race (e.g., qualified, charity), start wave assignment, and date and time they were leaving after the marathon. Collectively, we used these screening survey questions to identify inclusion/exclusion criteria, identify an individual’s pre-existing risk factors for GI injury (e.g., previous EHS), or help describe an individual’s response to the marathon (e.g., supplement use, recent illness).

#### Baseline survey

The baseline survey was delivered through Qualtrics or administered in paper format for participants recruited on-site at the pre-race expo. Seven questions characterized the participants’ race history, how they travelled to Boston or whether they lived in the city, and their goal pace or finish time for the marathon. Five questions asked about illness within the last 5 days. If the participant reported they were ill, a follow up question asked what symptoms they were experiencing. Finally, two questions characterized the sleep they had the previous night.

#### Pre-race, post-race, and 24-h post-race survey

Participants were asked four questions regarding non-steroidal anti-inflammatory drug (NSAID) use the night before and morning of the race, sleep quality (above average, average, below average) and quantity (<5, 6–7, 8–9, >9 h) the night before the race, and if they had taken any energy or electrolyte supplements the morning of the race. The post-race survey asked if participants completed the race and characterized events during the race such as if NSAIDs were taken, how often they stopped at water stations and type of fluids they consumed, if they consumed food and/or supplements during the race and what type, and if they experienced a medical event. For participants who took NSAIDs, a follow up question included type and dose of NSAID taken. Participants who experienced a medical event were asked the type of medical event and what mile the event occurred. The 24-h post-race survey asked if participants had taken any NSAIDs since finishing the race and about post-race sleep quantity and quality.

#### GI symptoms

Gastrointestinal symptoms were assessed using a questionnaire adopted from previous research (Pfeiffer et al., 2009; Pfeiffer et al., 2012). The index is divided into 3 sections: 1) upper abdominal problems (reflux/heart burn, belching, bloating, stomach pain/cramps, vomiting, nausea); 2) lower abdominal problems (intestinal cramps, flatulence, urge to defecate, left and right abdominal pain/stitch, loose stool, diarrhea); and 3) systemic problems (dizziness, headache, muscle cramps, urge to urinate, thirsty, fever, hands swollen, feet swollen, tired/fatigued, muscle soreness/weakness, tingling in arms, tingling in legs) (Pfeiffer et al., 2009). Symptoms are scored on a 10-point scale (0 = no problems at all and 9 = the worst it has ever been). A score of >4 is considered “serious” (Pfeiffer et al., 2012). Immediately pre-race, participants reported any symptom(s) they were currently experiencing. Post-race, participants were asked to recall symptoms they experienced during the race. At 24-h post-race, participants were asked to recall symptoms since completing the marathon.

#### Diet and activity logs

Participants tracked diet and physical activity for 3 days using an online nutrition software (FoodProdigy™, ESHA Research, Salem, OR). The 3 days included the day before the race (Sunday), day of the race (Monday), and the day after the race (Tuesday).

#### Urine specific gravity

Hydration status was characterized by urine specific gravity (Usg). Urine samples were collected pre-race, post-race, and 24-h post-marathon. At each time point, participants were provided a sealed urine sample cup and instructed to go to a nearby restroom to collect the sample. We measured Usg using a clinical refractometer according to the manufacturer’s instructions (Master-Sur, Atago company Ltd., Tokyo, Japan). A research assistant measured each sample in duplicate, with the average of the 2 measures being used for analysis. Before use, the refractometer was calibrated with distilled water. Samples were analyzed immediately after collection. Hydrated was defined as a Usg <1.025 and hypohydrated was defined as a Usg ≥1.025 (Armstrong et al., 2010). Urine specific gravity is moderately correlated with plasma osmolality (Popowski et al., 2001) and is a practical tool to assess hydration status in a field setting.

#### Risk factors

We reviewed self-reported information from the screening, baseline, pre-race, post-race, 24-h post-race surveys, and FoodProdigy logs to identify potential factors that may increase a participant’s risk for GI disturbances. Based on previous literature (Jeukendrup et al., 2000; De Oliveira et al., 2014), risk factors included: previous history of EHI (Stearns et al., 2020), alcohol consumption (Bishehsari et al., 2017), hydration (Van Nieuwenhoven et al., 2000), sleep quality and quantity (Patel et al., 2016), prescription or OTC medication and/or supplement use (Katzung, 2007), NSAID use (Lambert et al., 2007; Van Wijck et al., 2012a; McKenna et al., 2023), illness ≤5 days pre-race (Nieman et al., 1990), and how and when they travelled to the race or if they resided in Boston (Mues et al., 2014).

#### Intestinal cell injury

To measure intestinal cell injury, we chose intestinal fatty-acid-binding protein-2 (FABP2/I-FABP) because it correlates with splanchnic hypoperfusion (Van Wijck et al., 2011a; Van Wijck et al., 2012a). Recent attention on I-FABP has suggested this is a valid, sensitive marker for assessing human ischemia-reperfusion intestinal cell injury or damage following exercise (Van Wijck et al., 2011b; Van Wijck et al., 2012a; Van Wijck et al., 2013b; Morrison et al., 2014; Costa et al., 2017b; March et al., 2017; Snipe et al., 2018). Found in mature enterocytes of the small intestinal villi, I-FABP is a 14 kDa cytosolic protein that is released upon compromised cell membrane integrity. Subsequently, I-FABP enters the systemic circulation, making it a sensitive, acute marker of intestinal enterocyte injury (Kanda et al., 1996; Derikx et al., 2007a; Derikx et al., 2007b; Derikx et al., 2010). Although it is not a direct marker of compromised permeability, the intestinal villi undergo exercise-induced ischemia that results in loss of intestinal cell integrity leading to I-FABP being released (Ishimura et al., 2013).

A human FABP2/I-FABP Quantikine ELISA Kit (R&D Systems) was conducted according to the manufacturers’ instructions to assess participants’ intestinal cell injury. Sample analysis was conducted on the same plate to decrease inter-assay variation. All blood samples were collected post-race and 24-h post-race. Unfortunately, due to inclement weather, pre-blood samples could not be safely collected. Blood was collected from the antecubital vein into a 10 mL serum separator vacutainer tube (BD, Franklin Lakes, NJ), inverted several times to mix, left to clot for 30 min at room temperature, centrifuged at 3000 rpm for 15 min, serum pipetted into microtubes, and stored at −20°C until analysis. We report intestinal cell injury as absolute (in pg/ml). Due to large interparticipant variability in I-FABP, we also report percent change in intestinal cell injury (%ICI) from post-race to 24-h post-race for each participant (Van Wijck et al., 2013; Van Wijck et al., 2014; March et al., 2017).

### Experimental procedures

Following HHQ screening, eligible participants were sent an informed consent via Qualtrics and offered a follow-up phone call with a researcher to address any questions. Consenting participants were sent a “confirmed enrollment email” to participate. Instructions for logging food and physical activity and pre-race data collection were sent 1 week before the race. Participants were instructed to log their dietary intake the day before, day of the race, and anything consumed prior to their 24-h visit. A baseline survey was emailed 2 days before the race with reminders sent 1 day before the race to participants who had not completed the survey yet. Researchers verified all participants completed the pre-race survey prior to race day.

Pre-race data collection occurred before the participants’ scheduled wave start time. Start times ranged from 10:02 to 11:15 a.m. Upon arriving, participants provided a urine sample. Due to extreme thunderstorms, the GI symptoms index and pre-race survey were administered verbally and recorded by researchers rather than the participant completing paper surveys. Participants were reminded to meet at medical tent A immediately after the race, where a blood and urine sample were collected, and participants completed the GI symptom index and post-race survey. Participants then scheduled their 24-h visit and an informational card with directions and the appointment time was provided. Participants reported between 6:00 a.m. to 10:00 a.m. to provide a blood and urine sample and complete the GI symptom index and 24-h post-race survey.

### Data analysis

All data are presented as Mean ± Standard Deviation (SD). Changes in Usg and GI symptom scores were analyzed using 1 × 3 repeated measures ANOVAs. When appropriate, follow up analyses were conducted using paired samples t-tests. Differences in I-FABP were analyzed using paired samples t-tests. Associations between continuous variables were examined using Pearson product moment correlations. Chi-square tests of independence were conducted between overall mean GI symptom scores at pre-race, post-race, and 24-h post-race; mean upper, lower, and systemic GI symptoms at pre-race, post-race, and 24-h post-race by each risk factor (e.g., hydrated or hypohydrated). Appropriate sample size was assessed by expected cell frequency count. When expected cell counts were violated (<5), indicating an inappropriate sample size, Fisher’s exact test is reported. For all analyses, the ɑ level was set at .05. All statistical analyses were completed using R version 4.1.1. Post-hoc power analyses were calculated using G*Power version 3.1 (Dusseldorf, North Rhine-Westphalia, Germany) (Faul et al., 2009).

## Results

A total of 123 runners consented and 13 were excluded based on eligibility criteria. Seventy-four participants completed the baseline survey and 34 were removed from the sample as they did not have complete datasets (e.g., did not attend their 24-h post-race session). The final analysis included 40 runners. Post-hoc power analyses indicated that 40 subjects exceeds 80% power for detecting all relevant effects at an alpha level <0.05. Specifically, the sample size of 40 provided power to detect effect sizes as small as *f* ^*2*^ = .21 for the anova models and a *w* = .58 for the chi-square tests. Demographics are presented in Table 1. Males’ weight, height, and finish time were significantly different than females (Table 1). Table 2 presents the mean and ranges for environmental conditions during the race. Mean Usg at each time point for all participants is previously published (Atkins et al., 2022). Usg was significantly different across the 3 time points (F (2, 38) = 11.6, *p* < .001), with pre-race significantly lower than post-race (*p* < .001) and 24-h post-race (*p* < .001).

**TABLE 1:** Baseline Participant Demographics (mean ± standard deviation) and Risk Factors Reported [n (%)].

	Overall (*n* = 40, 100%)	Male (*n* = 19, 47.5%)	Female (*n* = 21, 52.5%)
Age (years)	44.9 ± 10.6	44.9 ± 10.8	44.8 ± 10.6
Weight (kg)	65.6 ± 11.8	73.7 ± 10.7^a^	58.7 ± 6.8
Height (cm)	171.5 ± 10.8	178.1 ± 9.0^b^	165.5 ± 8.6
Goal Finish Time (minutes)	211.8 ± 28.8	194.5 ± 19.0	227.4 ± 27.5
Actual Finish Time (minutes)	224.2 ± 32.0	205.8 ± 22.7^c^	240.9 ± 30.3
Calculated Race Pace (miles/hour)	8.5 ± 1.2	7.8 ± 0.8	9.2 ± 1.2
Travel to race			
Live in Boston	6 (15)	4 (21.1)	2 (9.5)
Car	6 (15)	5 (26.3)	1 (4.8)
Airplane	28 (70)	10 (52.6)	18 (85.7)
Days arriving before the race			
Did not travel	6 (15)	4 (21.1)	2 (9.5)
1 day	5 (12.5)	2 (10.5)	3 (14.3)
2 days	10 (25)	6 (31.6)	4 (19)
3 days	14 (35)	5 (26.3)	9 (42.9)
>4 days	5 (12.5)	2 (10.5)	3 (14.3)
Number of marathons completed prior to this marathon			
1 to 5	7 (17.5)	2 (10.5)	5 (23.8)
6 to 10	14 (35)	6 (31.6)	8 (38.1)
11 to 20	15 (37.5)	8 (42.1)	7 (33.3)
21 to 30	3 (7.5)	3 (15.8)	0 0)
>30	1 (2.5)	0 (0)	1 (4.8)
Believe they are heat acclimatized			
Yes	17 (42.5)	9 (47.4)	8 (38.1)
No	23 (57.5)	10 (52.6)	13 (61.9)
Ever become ill from exercising in the heat			
Yes	3 (7.5)	0 0)	3 (14.3)
No	37 (92.5)	19 (100)	18 (85.7)
Ever experienced exertional heat exhaustion			
Yes	6 (15)	3 (15.8)	3 (14.3)
No	34 (85)	16 (84.2)	18 (85.7)
Ever experienced exertional heat stroke			
Yes	0 (0)	0 (0)	0 (0)
No	40 (100)	19 (100)	21 (100)
Currently taking any prescription medications			
Yes	14 (35)	7 (36.8)	7 (33.3)
No	26 (65)	12 (63.2)	14 (66.7)
Currently taking any over the counter medications			
Yes	6 (15)	3 (15.8)	3 (14.3)
No	34 (85)	16 (84.2)	18 (85.7)
Currently taking any supplements			
Yes	30 (75)	13 (68.4)	17 (81)
No	10 (25)	6 (31.6)	4 (19)
Experienced any illness in previous 5 days before race			
Yes	5 (12.5)	2 (10.5)	3 (14.3)
No	35 (87.5)	17 (89.5)	18 (42.5)

Abbreviations: kg, kilograms; cm, centimeters.

^a^ Males significantly greater weight [t (38) = 5.545, *p* < .001, *d* = 1.322] than females.

^b^ Males significantly greater height [t (38) = 4.556, *p* < .001, *d* = 1.175] than females.

^c^ Male finish time significantly lower [t (38) = −4.254, *p* < .001, *d* = 1.123] than females.

**TABLE 2:** Environment.

	Mean ± standard deviation	Minimum–Maximum
Wet bulb globe temperature	18.4 ± 2.3	16.1–22.1
Relative humidity	66.5 ± 11.4	51.2–85.9

Note: Environmental monitoring occurred between 11:00 a.m.–4:25 p.m. Maximum wet bulb globe temperature and maximum heat index was recorded at 1:43 p.m. Maximum relative humidity was recorded at 4:25 p.m.

### Intestinal cell injury and risk factors

**FIGURE 1:**
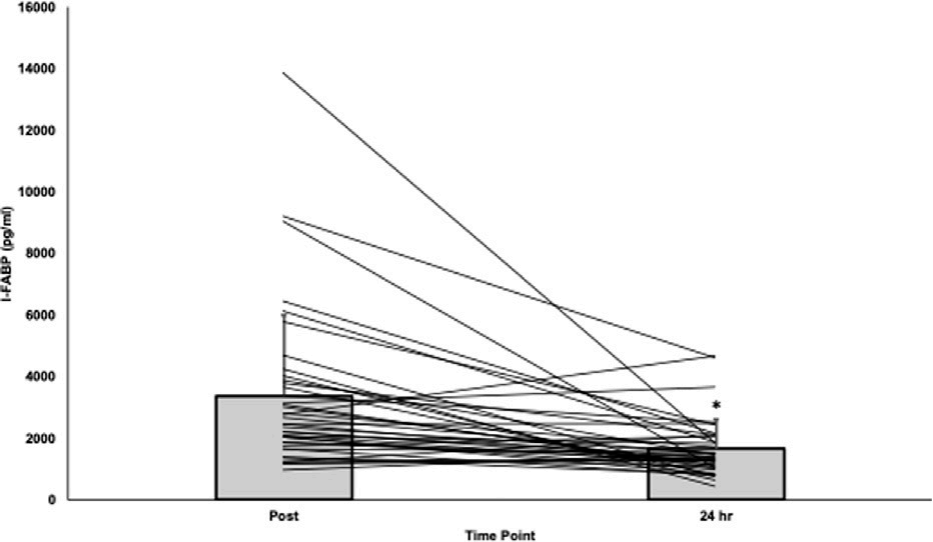
Absolute I-FABP post-race and 24-hours post-race. *I-FABP significantly decreased from post- to 24-h post-race [t (39) = −4.2, *p* < .001, d = −.669]. Abbreviations: I-FABP= intestinal fatty acid binding protein.

Overall, participants had significantly higher absolute I-FABP post-race (3367.5 ± 2633.5 pg/mL) compared to 24-h post-race (1657.3 ± 950.7 pg/mL, t (39) = −4.3, *p* < .001, d = −.669; Figure 1). The average percent change in I-FABP was −33.8% ± 41.6% (range: −90.0% to +71.2%).

No relationships (*p* > .05) or correlations were found at any time point between absolute I-FABP or %ICI to calories or macronutrients (carbohydrates, protein, fat) for individual days or combined days. Supplementary Table S1 presents the macronutrient intake the day before, day of, and day after the race. Supplementary Figures S1A–E displays food and beverage intake during the race. Supplementary Table S2 shows each risk factor and the results of the independent samples t-tests with post-race and 24-h post-race I-FABP and %ICI. Participants reporting no previous history of EHI had greater absolute mean post-race I-FABP (n = 32; 3675.4 pg/mL) compared to runners who reported a previous EHI (n = 8; 2135.9 pg/mL, *p* = .023). All other independent samples t-tests for risk factor presence for I-FABP post-race and 24-h post-race and %ICI were non-significant (*p* > .05). There was no statistically significant difference in %ICI and the number of risk factors present (i.e., one to two risk factors, three to four, or five or more). There were no significant correlations for dichotomous risk factors to I-FABP immediately post-race, I-FABP 24-h post-race or %ICI. Supplementary Table S3 displays absolute and %ICI, GI symptoms experienced, and risk factors present for each subject.

### GI symptoms and risk factors

Maximum, mean ± SD, overall incidence and percentage, and “serious” incidence and percentage at pre-race, post-race, and 24-h post-race is presented in Supplementary Table S4. There was a significant difference in overall GI symptom scores across each of the 3 time points (F (2, 38) = 41.37, *p* < .001), with the highest average score at post-race (.84 ± .68) compared to pre-race (.09 ± .12) and 24-h post-race (.44 ± .28).

**FIGURE 2:**
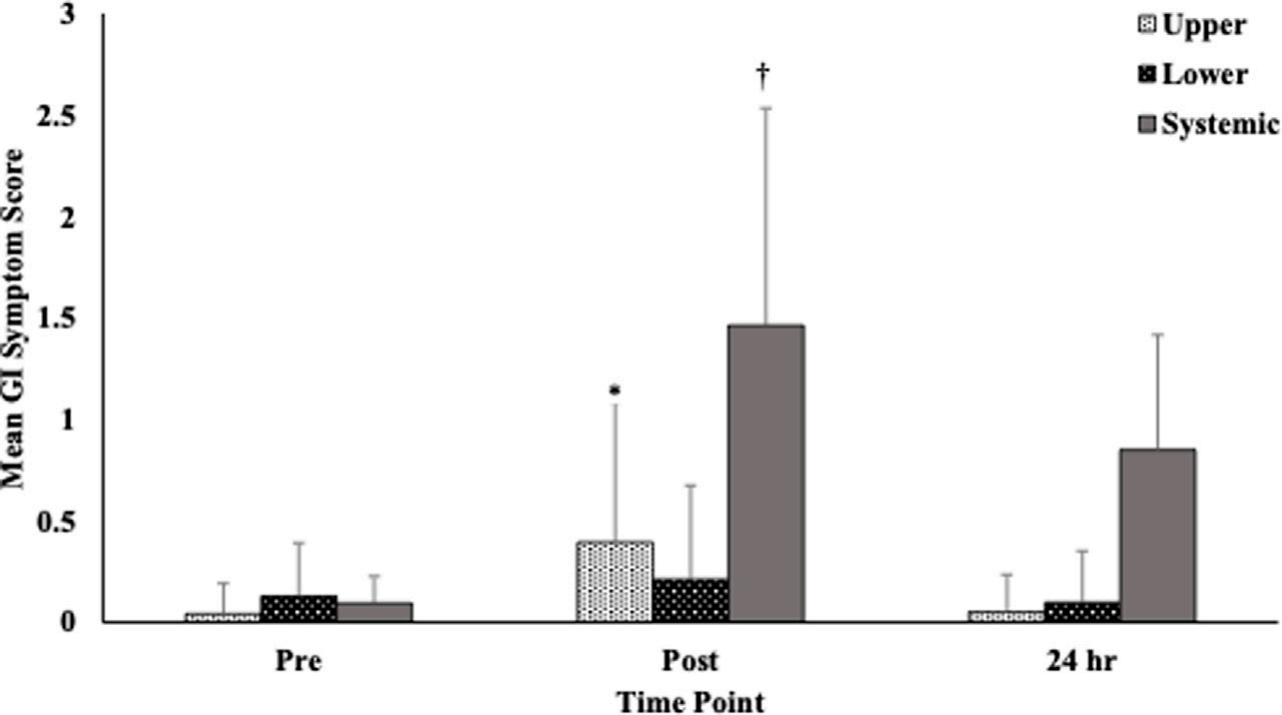
Gastrointestinal symptoms by location (upper, lower. systemic) at each time point. Notes: Symptoms are divided into 3 sections (upper abdominal problems, lower abdominal problems, systemic problems) and are scored on a 10-point scale (0 = no problems at all and 9 = worst it has ever been; >4=“serious”). Responses for each section and time point were averaged before analyzed. *Upper GI symptom scores were significantly higher at immediately post-, compared to pre- (Meandiff = 0.35, *p* = .001) and 24-hours post-race (Meandiff = 0.34, *p* < .001). ^†^Systemic symptoms were significantly different at all time points with the post- measure having the highest symptom score. Abbreviations: GI = gastrointestinal.

Given the varied nature of GI symptoms that might be present, we also investigated how symptoms associated with the upper/lower GI and those that might be considered systemic in nature were affected over the measurement period. There were no significant differences observed in symptom severity across time points when comparing serious (score >4) and non-serious symptoms. There were, however, significant differences observed when focusing on both upper and systemic symptoms (Figure 2).

Intestinal cell injury post-race was positively correlated with GI symptom scores post-race (r = .31, *p* = .048). Higher post-race I-FABP values were associated with greater overall post-race GI symptoms. Post-race Usg was also significantly correlated with GI symptom post-race scores (r = .33, *p* = .041). Pre-race GI symptom scores (r = .32, *p* = .046) and pre-race Usg (r = .33, *p* = .03) were directly correlated with GI symptom scores at 24-h post-race. Higher Usg values at pre-race were associated with a greater incidence of upper GI symptoms (r = .33, *p* = .038) as well as a greater incidence of systemic symptoms 24-h post-race (r = .40, *p* = .012). Post-race Usg was significantly associated with post-race lower GI symptoms (r = .37, *p* = .026).

The more risk factors present, the more likely to report systemic GI symptoms pre-race (r = .45, *p* = .004). This analysis also observed significant inverse correlations between the average 3-day calories and post-race upper GI symptoms (r = −.32, *p* = .044) and post-race lower GI symptoms (r = −.34, *p* = .035).

Participants reporting any pre-race symptoms (χ^2^ (1) = 4.912, *p* = .046, Cramer’s V = .350, OR = 9.0, 95% CI = .989–81.929), specifically pre-race lower GI symptoms (χ^2^ (1) = 6.144, *p* = .025, Cramer’s V = .392, OR 7.222, 95% CI 1.340–38.917) were less likely to take NSAIDs after the race, as indicated by the 24-h post-race survey. Those reporting upper GI symptoms at pre-race were more likely to report experiencing a medical event during the race (χ^2^ (1) = 8.254, *p* = .011, Cramer’s V = .454) and upper GI symptoms at post-race (χ^2^ (1) = 4.912, *p* = .042, Cramer’s V = .350). Participants reporting pre-race lower GI symptoms were more likely to reside in Boston (χ^2^ (1) = 5.431, *p* = .039, Cramer’s V = .368, OR .130, 95% CI .020–.858) and more likely to take NSAIDs after the race (χ^2^ (1) = 6.144, *p* = .025, Cramer’s V = .392, OR 7.222, 95% CI 1.340–38.917), as indicated by the 24-h post-race survey.

Participants reporting any post-race GI symptoms were less likely to have taken NSAIDs the night before the race (χ^2^ (1) = 11.930, *p* = .019, Cramer’s V = .546). Participants reporting any post-race GI symptoms were more likely to report 24-h post-race GI symptoms (χ^2^ (1) = 19.487, *p* = .05, Cramer’s V = .698). Specifically, 24-h post-race systemic symptoms were more likely reported by those with post-race GI symptoms (χ^2^ (1) = 19.487, *p* = .05, Cramer’s V = .698). Lower 24-h post-race GI symptoms were reported more often with participants who reported taking OTC medications (χ^2^ (1) = 6.016, *p* = .042, Cramer’s V = .403, OR = 9.333, 95% CI = 1.270–68.597).

## Discussion

The current study assessed GI epithelial barrier integrity and the incidence and severity of numerous GI symptoms on race day and the day after the race, as well as explored potential predictive factors of GI disturbances associated with running a marathon. Overall, our study continues to support existing literature that marathon runners experience GI disturbances (Keeffe et al., 1984; Hölmich et al., 1988; Riddoch and Trinick, 1988; Halvorsen et al., 1990; Pugh et al., 2018; Pugh et al., 2019; Walter et al., 2021). Although pre-race intestinal cell injury was not measured, an overall decrease in I-FABP from post-race to 24-h post-race indicates some recovery from the epithelial damage induced from running the marathon. Unfortunately, extrapolating more about recovery without pre-race measures is not possible. However, based on subjective GI symptom scores increasing from pre-race to post-race and decreasing from post-race to 24-h post-race, it appears participants’ GI disturbances from running the marathon were improving.

### Intestinal cell injury

Normal healthy resting I-FABP in adults is ≤200 pg/mL (Funaoka et al., 2011). Values for our participants after running ranged from 429.6 to 14086.6 pg/mL. Our average I-FABP values immediate post-race (3367.5 ± 2633.5 pg/mL) and 24-h post-race (1657.3 ± 950.7 pg/mL) are slightly higher than marathon runners in Walter et al. (2021) (post = 2593 ± 1373 pg/mL and 24-h post = 1086 ± 302 pg/mL). The decreases from post-race to 24-h post-race are similar between Walter et al. (2021) and our study; though, without pre-race values we cannot determine whether a participant reached a baseline or “normal” value. For reference, in Walter et al. (2021) pre-marathon I-FABP was 1129 ± 493 pg/mL. We can say that 77.5% of our participants showed a negative %ICI value, indicating participants were recovering, to some extent, from the GI damage induced by running the marathon. In another field study using I-FABP, Zadow et al. (2020) assessed intestinal cell injury 24-h before and immediately after a marathon between runners wearing and not wearing compression socks. Although Zadow et al. (2020) did not report mean values, I-FABP increased from 24-h pre-race to immediately post-race. Pre-race values were ∼1000 pg/mL or less and the highest post-race value was ∼5,000 pg/mL. Interestingly, those in the control group running at slower rates had higher I-FABP levels; the authors suggested this was related to absolute intensity and that significant GI damage only occurs at the individual’s intensity threshold. Unfortunately, the authors did not conduct any further follow-up after the race to examine recovery.

The high I-FABP values among some of our participants are similar to clinical measures found in patients with GI disease (Derikx et al., 2007a; Derikx et al., 2008; Wiercinska-Drapalo et al., 2008; Adriaanse et al., 2016; Abdel-Haie et al., 2017; Prendergast et al., 2017; Hundscheid et al., 2020), as well as those suffering from exercise-associated collapse (EAC) in Walter et al. (2021). Compared to no EAC (control), runners suffering from EAC showed significantly higher I-FABP levels (post-EAC = 15389 ± 8,547 pg/mL; *n* = 8), which persisted 1 h after the collapse (13951 ± 10476 pg/mL; *n* = 3). None of our participants experienced EAC or a serious medical event that prevented them from completing the marathon. Our higher I-FABP levels may in part be explained by the more strenuous Boston Marathon course and our runners undergoing greater environmental strain. Marathon routes in both Walter et al. (2021) and Zadow et al. (2020) are flat, fast courses, often recommended to individuals trying to obtain a personal best. On the other hand, the Boston Marathon is a mostly downhill, slower course with two steep up-hills toward the latter half of the route. More eccentric load and having to work harder to maintain a pace/goal time creates greater metabolic heat and, thus, for that individual nearing their threshold, greater GI damage. This is supported by Walter et al. (2021) who, despite the cool environmental temperature (start and finish = 8°C), the average core body temperature at the time of collapse in the EAC group = 39.7°C, compared to 36.2°C in the control group post-race. Not only did those experiencing EAC exhibit significantly greater GI damage, EAC runners were significantly more hyperthermic. Although this does not tell us whether high core body temperature or GI damage “triggered” the EAC, it does support the complex integration of the GI tract in a runner developing EHI. In Zadow et al. (2020), the mean environment (16.4°C) was similar to our study (18.4°C); their I-FABP levels were lower, which, again may be due to the course and absolute intensity the runners were competing at during the Boston Marathon.

There appears to be a dose-response with intensity and GI distress, with damage occurring if the activity is at least 70% VO_2max_ and prolonged (>60 min) (Mohr et al., 2020; Edwards et al., 2021; McKenna et al., 2022). Several laboratory studies support the relationship between intestinal cell injury changes and various intensity, activity type, and environmental strain. In a lab-simulated marathon on an outdoor track (16°C–17°C), I-FABP increased from pre-race (460 ± 221 pg/mL) to post-race (1392 ± 867 pg/mL), but at 1-h post-marathon, the I-FABP levels were not significantly different from pre-race (Pugh et al., 2019). For shorter exercise bouts, 2 h of running (23°C ± 1°C) at 60% VO_2max_ followed by a 1-h self-paced running distance test (24°C ± 1°C) resulted in mean I-FABP concentrations >1000 pg/mL (Costa et al., 2017a). Following two 1-h treadmill running bouts (65% VO_2max_) under a normoxic and hypoxic environment, I-FABP only significantly increased in the hypoxic group by 68% post-run, returning to baseline at 1-h (Hill et al., 2020). The aforementioned, tightly controlled, laboratory studies in healthy subjects undergoing an endurance stimulus report I-FABP values lower than ours and participants returning to below baseline values 1-h post-exercise (Van Wijck et al., 2012b; Pugh et al., 2019; Hill et al., 2020). It is not surprising that our I-FABP values would be higher after more than 3 h of intense running and based on previous research, we assume our participants’ intensity to be >70% VO_2max_ (Sjödin and Svedenhag, 1985; Billat et al., 2001; Legaz-Arrese et al., 2007). Further, while we aimed to control many confounding variables (e.g., fitness level, medical history), as a field study, we were not able to control other factors (e.g., hydration status, nutritional intake) that could influence our higher values.

### GI symptoms

It is well established endurance athletes experience GI symptoms during and after running (Keeffe et al., 1984; Hölmich et al., 1988; Riddoch and Trinick, 1988; Halvorsen et al., 1990; Costa et al., 2017a; Pugh et al., 2018). Symptom incidence and severity generally increases with activity intensity (Keeffe et al., 1984; Riddoch and Trinick, 1988; Edwards et al., 2021) and symptoms are more often reported by those who have experienced GI symptoms in the past (Pfeiffer et al., 2009; 2012). Symptoms vary in severity from mild discomfort (e.g., heartburn) to severe (e.g., bloody diarrhea) and also vary greatly within and between individuals. GI symptoms are a concern because they can reduce the ability to maintain running speed. In extreme cases, GI symptoms may lead the runner to withdraw from events (Pugh et al., 2019) and should serve as a warning sign, as severe life-threatening GI blood loss has been reported (Thompson et al., 1982; Heer et al., 1987; Lucas and Schroy, 1998).

As expected, our mean pre-race GI symptom scores were lower than both post-race and 24-h post-race. Symptoms the morning after the race were lower than post-race, suggesting exercise-induced disturbances improved. The idea of the runners recovering may seem contradictory to our results, with the overall frequency of GI symptoms reported being greater at 24-h post-race (97.5%) than immediately post-race (95.0%). Symptoms are characterized by location: upper (e.g., belching, bloating) or lower (e.g., flatulence, diarrhea). For our study, and based on previous studies (Pfeiffer et al., 2009; Pfeiffer et al., 2012), we included systemic symptoms (e.g., headache, fatigue) in our questionnaire. These systemic symptoms are not necessarily GI related but affect a runner’s performance and may indicate a medical related issue. For each of our time points, symptoms were predominantly systemic and included feelings of tiredness/weakness, thirst, and muscle soreness. Hence, although the overall percentage of individuals experiencing symptoms at 24-h post-race would indicate runners’ GI disturbance did not improve, these symptoms were predominately systemic. This notion is supported by the decreasing frequency of both upper GI (15.1% to 3.5%) and lower GI (10.8% to 8.4%) symptoms post-race to the morning after. Meanwhile, systemic incidence increased from post-race to 24-h (74% to 88%).

Specific to upper and lower GI symptoms, at pre-race, our participants most frequently reported lower symptoms, including loose stool and urge to defecate. At post-race, the most common upper GI symptoms included nausea, stomach pain/cramps, and belching. Finally, at 24-h post-race, the most frequently reported GI symptom was loose stool (lower). Our results are similar to previous studies, which consistently show the top reported lower GI symptoms are diarrhea/loose stool and urge to defecate (Riddoch and Trinick, 1988; Halvorsen et al., 1990), followed by flatulence/gas (Riddoch and Trinick, 1988; Halvorsen et al., 1990; Pugh et al., 2018). For upper symptoms, nausea (Keeffe et al., 1984; Halvorsen et al., 1990; Pugh et al., 2018; Etxebarria et al., 2021), belching (Pugh et al., 2018), and stomach pain/cramping (Halvorsen et al., 1990) are most commonly reported.

Whether it is due to a performance or health concern, the potential consequences of unmitigated bacterial translocation warrants continued examination of the relationship between intestinal cell injury and symptomatology. Several possible mechanisms exist that link GI symptoms and I-FABP. First, symptoms may be in response to increased circulating endotoxin and/or the inflammatory response to damaged epithelial cells (Hill et al., 2020). Second, the ischemic environment that follows splanchnic vasoconstriction during exercise is associated with increased GI permeability (Van Wijck et al., 2012b) and nausea, vomiting, and diarrhea (De Oliveira and Burini, 2009; 2011). Third, the mechanical jarring during running is shown to increase GI permeability and elicit diarrhea, urge to defecate, and flatulence. Speculatively, symptom occurrence and severity may be due to a combination of mechanisms. For example, cramping and blood loss in stool is likely due to mechanical trauma and ischemia combined (de Oliveira et al., 2014). Our study cannot definitively state which mechanism is involved but supports a link between GI symptoms and I-FABP based on the positive association between post-race I-FABP and post-race GI symptoms. The greater the I-FABP, the more likely individuals were to report GI symptoms.

### Risk factors affecting intestinal cell injury and GI symptoms

The individual variation in exercise induced GI responses makes determining a “normal” for I-FABP or symptoms increasingly difficult. At the same time, it is important to continue to examine potential risk factors and better understand the impact on GI damage during exercise in various populations. We selected risk factors known to exacerbate GI barrier dysfunction and symptoms, which included current or recent illness or infection, pre-existing medical conditions, medication and/or supplement use, nutrition, sleep, and travel. For each risk factor, we briefly mention the mechanism(s) as to how these affect GI barrier function or elicit GI symptoms and what we identified in our population of healthy, well-trained marathon runners completing the Boston Marathon.

#### Recent illness, pre-existing medical conditions, and medications

Only 5 participants reported experiencing an illness within 5 days of the marathon and this was not associated with greater I-FABP or symptoms. Beginning exercise with an illness or infection means the immune system is already recruited to neutralize the present pathogen, and this response may last several days with or without symptoms present. An individual exercising with an active immune system response has a compromised ability to neutralize endotoxin in systemic circulation (Lim and Mackinnon, 2006; Selkirk et al., 2008). The commonality between illness symptoms (e.g., fever, diarrhea, and vomiting) and GI symptoms among endurance runners is due to the inflammatory response elicited during both states.

No participants had a previous history of EHS, which is important to note because the systemic inflammatory response and organ damage that occurs during EHS can elicit long-term physiological changes that prevent the person from effectively thermoregulating during activity. These adaptations can last for months or years after the incident (Lim and Mackinnon, 2006; McDermott et al., 2007; Casa et al., 2015). Clinically, these long-term consequences are not seen among individuals who have experienced exertional heat exhaustion. We had 8 participants report previously experiencing EHI, and interestingly, these runners exhibited lower absolute I-FABP levels post-marathon. We cannot speculate why those reporting previous EHI had lower post-marathon absolute intestinal cell injury, especially considering the %ICI change from post-to 24-h post-marathon was not significant (Supplementary Table S3). A closer examination of these 8 runners showed 87.5% were taking a supplement, 75% travelled to the race, 75% were not heat-acclimatized, and 62.5% took a prescription medication. Presumably, having one, or a combination of factors, would more likely promote GI damage and higher I-FABP. Without measuring core temperature, heat shock proteins (HSP), or immune markers, we cannot definitively state why participants with previous EHI contradictorily exhibited lower mean post-race I-FABP.

The most frequently reported prescriptions taken by our participants included antidepressants, anticholinergics, and antihistamines. Allergy/asthma (e.g., antihistamines) and NSAIDs were the most commonly reported OTC medications. Mental health, allergy/asthma, and NSAID medications are associated with altering sweat production or blood flow, which can lead to thermoregulatory impairment and promote greater heat storage (Kalisch Ellett et al., 2016). Higher GI temperatures indirectly disrupt or exacerbate exercise induced GI barrier damage. Further, despite being an anti-inflammatory, NSAIDs directly damage the GI tract (i.e., increase GI permeability) and elicit GI side effects (e.g., bleeding, mucosal ulcers) at rest and following exercise (Van Wijck et al., 2012b; McKenna et al., 2023). Although the number of our participants taking medications were limited and we did not find a relationship with intestinal cell injury, we did identify those reporting pre-race GI symptoms were less likely to take NSAIDs post-race. Possibly, running the marathon exacerbated pre-race symptoms, making them less inclined to take medications that could intensify symptoms.

#### Nutrition and hydration

Based on the 3-day calorie average, the less calories our participants consumed the more likely they were to report post-race upper and lower GI symptoms. Extrapolating why is difficult considering GI symptoms were not associated with calories on individual days. We also did not find any caloric or macronutrient intake associations with intestinal cell injury measures. Runners may have been eating less because they already had GI symptoms, or they may have been attempting to prevent symptoms. Travel or race day anticipatory stress may have also contributed to reducing the runner’s appetite. Avoiding food and/or beverage intake to prevent GI disturbances is not recommended. Inadequate energy intake or inappropriate nutrient replenishment can be detrimental to performance, with prolonged nutrient deficits threatening homeostasis (e.g., reduced bone mineral density).

After combining food (e.g., oranges) and beverage (e.g., Gatorade) intake and estimating grams per hour of carbohydrates consumed during the race, our runners averaged ∼30.9 g per hour, which is on the lower end of the carbohydrate recommendations (30–60 g per hour) (American Dietetic Association et al., 2009). On the individual level, intakes ranged from 3.4 to 47.3 g per hour. On average, our runners were not consuming abnormally large amounts of carbohydrates during the race that would potentially promote GI discomfort.

The relationship between higher post-race Usg and post-race GI symptoms in our runners supports previous work showing hypohydration exacerbates GI disturbances. Exercising in a hypohydrated state reduces the blood volume required to adequately maintain cardiovascular function (e.g., heart rate) and dissipate heat (e.g., sweating). As heart rate and core temperature rise, there are further reductions in splanchnic blood flow. Thus, the ischemic-hypoxic environment in the GI tract is exacerbated, increasing the GI tissue susceptibility to damage and associated symptoms. Hypohydration is shown to exacerbate intestinal injury after exercising in both thermoneutral and thermal environments (Lambert et al., 2008; Costa et al., 2019). Costa et al. (2019) showed I-FABP increases of 166% in hypohydrated and only 86% in euhydrated runners. Although not significant, GI symptoms were reported by 82% of hypohydrated runners compared to 64% of euhydrated (Costa et al., 2019).

#### Travel and sleep

Mechanisms related to travel are not completely clear, but GI damage is believed to be related to the mental stress, new environment, and greater exposure to contaminated surfaces/people (Schwellnus et al., 2012; Mues et al., 2014). It was surprising to see participants who lived in and did not travel to Boston were more likely to experience pre-race lower GI symptoms. It is possible participants who lived in the area were more relaxed with their habits. People traveling, who were aware of the potential impact, may have made it a priority to adequately fuel or arrive early enough to familiarize themselves with the new sleeping conditions and time zone.

Based on Centers for Disease Control and Prevention (CDC) recommendations, adults aged 18–60 years, where our participants would be included, should obtain a minimum of 7 h of sleep (CDC, 2019). Although not statistically related to GI responses, 70% of our runners the night before the race and 62.5% the night after the race reported sleeping less than 7 h. When looking at how they perceived their sleep at any time point, only 22.5% reported sleeping a “below average” amount. Two of those 9 still reported “below average” sleep at 24-h post-race. Sleep plays an important role in normal physiological function of the GI tract. Sleep loss is shown to promote greater GI symptoms compared to groups with appropriate sleep (Cremonini et al., 2009). Circadian rhythm disruption plays a major role in GI microbiome health and GI barrier integrity (Codoñer-Franch and Gombert, 2018).

#### 24-Hours after the race

One of the unique aspects of our study was to examine what factors may affect the runner’s GI distress the day after the marathon. For the 22.5% of our participants who showed no change or an increase in %ICI from post-race to 24-h post-marathon, we did not identify any statistically significant factors to explain the increased intestinal cell injury at 24-h. We hypothesized post-race activities would inhibit recovery and/or further damge the GI barrier. For instance, alcohol directly disrupts the GI lining, increases GI symptoms (e.g., vomiting) (Bode and Bode, 1997; Bishehsari et al., 2017), and indirectly causes immune (Barr et al., 2016), cardiovascular (Bau et al., 2011), thermoregulatory (Yoda et al., 2005; Yoda et al., 2008), and sleep disruptions (Wilkinson et al., 2018). Contrary to our hypothesis, we did not find that consuming alcohol Monday after the race influenced absolute or %ICI change from post-race to 24-h post-race. We also did not find that absolute or %ICI change was influenced by receiving less than an average amount of sleep.

We did find some factors, specifically hydration status and using OTC medications, affected GI symptoms 24-h post-race. Runners with higher Usg (less hydrated) were more likely to report 24-h upper and systemic GI symptoms. It is possible being less hydrated pre-race would promote more GI distress during and after the race. If a runner experienced GI symptoms such as diarrhea or vomiting it would prevent them from fully rehydrating. Regarding systemic symptoms, this association is likely attributed to thirst being listed as a systemic GI symptom and reported by 50% of our runners at 24-h (Supplementary Table S4). While, speculatively, runners reporting more lower GI symptoms 24-h after the marathon following taking OTC medications may have prolonged the GI barrier recovery due to ingesting medications known to contribute to barrier damage (e.g., NSAIDs).

### Practical application

Although general recommendations are a starting point, they may not work for everyone. Identifying individual GI discomfort triggers and tolerance levels allows a more targeted, patient-centered approach to optimize health and performance. GI damage is a multifactorial issue, where trial and error may be necessary during a training protocol. The individual aspect cannot be stressed enough; what works for one runner may not work for another. Strategies need to be tailored to the individual and likely, patience will be necessary to best determine what may be causing GI symptoms. Sometimes, symptoms may be due to a single, specific situation (e.g., sleep loss) and the runner never experiences GI symptoms again. Other times, the runner may have multiple factors that contributed to their GI damage or they may experience prolonged symptoms. Chronic or worsening symptoms should serve as a warning sign that something is not right, exercise cessation should be considered and further examination into the cause is warranted. Documentation is a simple starting point to track symptoms and what strategies reduce or increase symptoms. Documentation should include events (e.g., 3 h of sleep the night before) and intake (i.e., food and fluid) leading up to the event where they experienced symptoms. Once potential causes are determined, trial and error can begin to pinpoint the triggers and hopefully mitigate the prevalence and severity of GI symptoms. The recommendations that follow focus on the risk factors previously discussed.

Some of the easiest risks to address include fluid, hydration, and training behaviors. For instance, runners should prioritize acclimatizing to environmental temperatures, particularly if temperatures are hotter at the event location than where they have been training. Runners should avoid introducing new nutritional strategies on the day of an event, only consuming foods and fluids they know have not caused issues in the past. Finally, nutrient intake and timing post-event should be prioritized to minimize fluid losses during exercise and replenish muscle glycogen rapidly. In the event runners are too symptomatic to consume solid food, liquids may be better and should be consumed within 2 h post-exercise to begin the nutrient recovery process. Ultimately, euhydration allows appropriate tissue perfusion and should be prioritized by all before, during, and after activity, especially in individuals prone to GI disturbances.

More difficult factors for a runner to control include recent illness, sleep loss, and medication use. Individuals should avoid exercise if they have a fever and be cautious in the days after their fever has ceased due to a heightened immune response. If an individual experiences diarrhea or vomiting from their illness, these will promote fluid losses and fluid intake should be prioritized. We include medication use as a less controllable factor because it would be naïve to expect an individual to discontinue using medication(s) vital for their overall health. Regardless of the lack of significant results regarding medications in this paper, based on previous studies showing medications negatively affect the GI tract (Kalisch Ellett et al., 2016), we continue to support the recommendation that individuals be aware of medications known to damage the GI tract or alter physiological function before, during, and after exercise. If a runner is concerned about their medication, they should consult with their physician and/or pharmacist to better understand the mechanism of action, side effects, strategies to mitigate adverse events, and potential treatment alternatives. Finally, sleep is often the most-overlooked priority in training, and runners should minimize circadian rhythm disruptions that may exacerbate GI disturbances by prioritizing at least 7 h of sleep a night. If possible, runners should arrive early to events in different time zones or adjust their sleep schedule prior to leaving in order to ensure adequate, quality sleep at the event location.

### Limitations and future research

A major limitation to our study is the inability to collect baseline (pre-race) intestinal cell injury measures. Despite the majority of participants’ intestinal cell injury measures decreasing 24-h post-race, without knowing their pre-race levels, it is hard to speculate about the amount of recovery. The lack of baseline intestinal cell injury limits us from identifying any participants who started the race with higher than “normal” I-FABP values. While we did screen individuals and exclude any with self-reported GI disease, it is possible individuals may have had higher than normal I-FABP to start if they had an undiagnosed, underlying GI condition. We also are unable to determine any association between the risk factors and baseline I-FABP measures. Future research should obtain baseline values as well as use individuals with and without GI disease to help establish the “normal” range of exercise induced I-FABP in participants.

Using self-reported questionnaires, we assume participants reported accurately and honestly. Several of our surveys were used in an attempt to identify and control factors known to affect GI injury. However, we acknowledge that asking participants to self-report introduces recall bias, decreasing the accuracy of this information. One particular limitation is our choice to use a less validated GI symptom index rather than an instrument such as the Gastrointestinal Symptom Rating Scale (GSRS). In the future, using the GSRS would provide more reliable data as well as allow us to compare our results to other studies who have used this tool in marathon runners. Participants were asked to recall the symptoms they experienced during the race and over the hours after the race ended.

Because our study was limited to healthy individuals without pre-existing medical conditions and who were well-trained, the results may not be generalizable to individuals who have GI, metabolic, cardiovascular, inflammatory, or other medical conditions or who are less trained, as all of these factors can negatively affect GI responses during exercise. Finally, although we achieved appropriate statistical power overall, the small sample sizes within each risk factor category (e.g., taking NSAIDs vs. not taking NSAIDs) likely prevented us from finding associations or where significant associations occurred, limits the reliability of these findings.

Future research should continue to examine participants with known risk factors to compare groups and increase the sample size. Research is also warranted on different training levels (e.g., recreational vs. elite) and in different environmental conditions. Due to the lack of heart rate measurement, it was not possible to estimate the % VO_2max_ our participants were exercising at. More accurate indications of intensity, as well as adding core temperature, heart rate, and immune response measures in the future will help to determine the relationship between GI disturbances and other physiological confounding variables.

## Conclusion

Our study was unique in that we sought to take a comprehensive look at several risk factors and how these either independently or concurrently impacted GI responses in marathon runners. Another unique aspect to our study was examining intestinal cell injury and GI symptoms the day after the marathon to determine what factors may influence GI distress while runners recover. For healthy, well-trained marathon runners, we found both intestinal cell injury and the frequency of GI symptoms decreased from immediately after the marathon to the day after the marathon. This reduction in GI disturbances the day after the race was influenced by hydration status. The increased popularity of long-distance running and the high incidence of GI symptoms in this population warrants additional investigations that address mechanisms of intestinal cell injury and GI symptoms, while also considering the individuality of risk factors. Future research should continue focusing on educating clinicians and runners on strategies to prevent symptoms and mitigate potential health and performance consequences.

## Data Availability

The raw data supporting the conclusions of this article will be made available by the authors, without undue reservation.

## Supplementary material

TheSupplementary Material for this article can be found online at: https://www.frontiersin.org/articles/10.3389/fphys.2023.1268306/full#supplementary-material

SUPPLEMENTARY FIGURE S1 Food and beverage intake during the race. **(A)** Estimated amount of fluid consumed by runners during the race. **(B)** Percentage of stations runners stopped at for fluids during the race. **(C)** Types of food consumed during the race. **(D)** Total number of energy/supplements used during the race. **(D)** Type of energy/supplements used during the race.
