# Supplementary figures and images for: Gastrointestinal cell injury and perceived symptoms after running the Boston Marathon
Source: Front Physiol. 2023 Oct 16;14:1268306. doi: 10.3389/fphys.2023.1268306 (PMC10615131; doi:10.3389/fphys.2023.1268306)

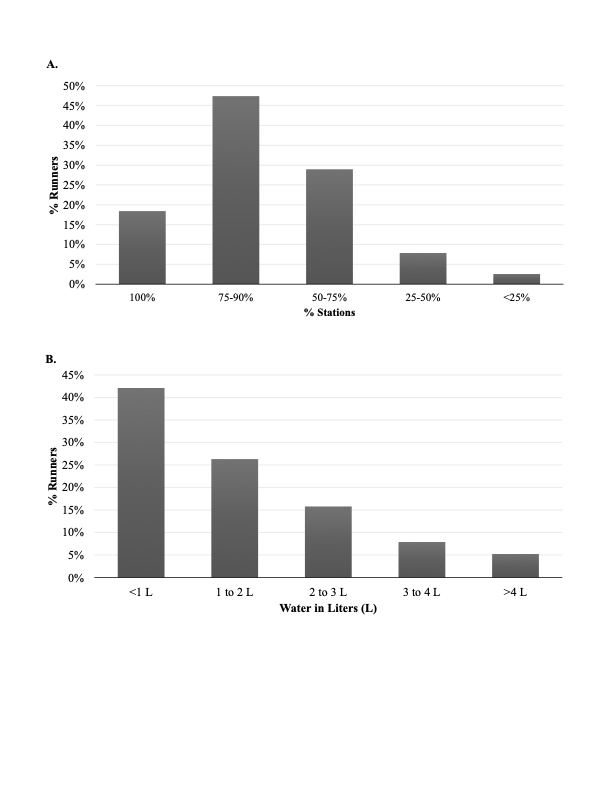

Supplement: Supplementary file 1 [file Image1.tiff]
