# Supplementary material for: Gastrointestinal cell injury and perceived symptoms after running the Boston Marathon
Source: Front Physiol. 2023 Oct 16;14:1268306. doi: 10.3389/fphys.2023.1268306 (PMC10615131; doi:10.3389/fphys.2023.1268306)
Supplement: Supplementary file 2 [file Table4.pdf]

Supplemental Table 4. Gastrointestinal symptom scores for pre-, post- and 24-hours post-race.

Symptom	Pre-				Post-				24-hours Post-			
	Max	M ± SD	Incidence n (%)		Max	M ± SD	Incidence n (%)		Max	M ± SD	Incidence n (%)	
			Overall	Serious			Overall	Serious			Overall	Serious
**Upper**												
Reflux/Heartburn	0	0.0 ± 0.0	0 (0)	0 (0)	1	0.0 ± 0.2	1 (2.5)	2 (5)	1	0.0 ± 0.2	1 (2.5)	0 (0)
Belching	0	0.0 ± 0.0	0 (0)	0 (0)	5	0.5 ± 1.1	9 (22.5)	2 (5)	2	0.1 ± 0.3	1 (2.5)	0 (0)
Bloating	3	0.1 ± 0.5	3 (7.5)	0 (0)	5	0.3 ± 1.0	3 (7.5)	1 (2.5)	0	0.0 ± 0.0	0 (0)	0 (0)
Stomach Pain/Cramps	2	0.1 ± 0.3	2 (5)	0 (0)	8	0.8 ± 1.7	9 (22.5)	2 (5)	5	0.1 ± 0.8	1 (2.5)	1 (2.5)
Vomiting	0	0.0 ± 0.0	0 (0)	0 (0)	1	0.0 ± 0.2	1 (2.5)	0 (0)	0	0.0 ± 0.0	0 (0)	0 (0)
Nausea	2	0.1 ± 0.3	1 (2.5)	0 (0)	8	0.7 ± 1.7	9 (22.5)	2 (5)	3	0.1 ± 0.5	2 (5)	0 (0)
**Lower**												
Intestinal Cramps	2	0.1 ± 0.3	2 (5)	0 (0)	8	0.4 ± 1.4	6 (15)	1 (2.5)	5	0.2 ± 0.8	3 (7.5)	1 (2.5)
Flatulence	2	0.1 ± 0.3	1 (2.5)	0 (0)	2	0.1 ± 0.4	2 (5)	2 (5)	0	0.0 ± 0.0	0 (0)	0 (0)
Urge to Defecate	3	0.2 ± 0.7	4 (10)	0 (0)	7	0.2 ± 1.1	2 (5)	1 (2.5)	5	0.2 ± 0.8	2 (5)	1 (2.5)
L Abdominal Pain/Stitch	0	0.0 ± 0.0	0 (0)	0 (0)	3	0.3 ± 0.8	5 (12.5)	2 (5)	1	0.0 ± 0.2	1 (2.5)	0 (0)
R Abdominal Pain/Stitch	0	0.0 ± 0.0	0 (0)	0 (0)	8	0.4 ± 1.5	5 (12.5)	1 (2.5)	3	0.2 ± 0.7	2 (5)	0 (0)
Loose Stool	5	0.5 ± 1.3	7 (17.5)	2 (5)	1	0.0 ± 0.2	1 (2.5)	2 (5)	2	0.2 ± 0.5	4 (10)	0 (0)
Diarrhea	1	0.1 ± 0.2	2 (5)	0 (0)	0	0.0 ± 0.0	2 (5)	2 (5)	0	0.0 ± 0.0	0 (0)	0 (0)
**Systemic**												
Dizziness	0	0.0 ± 0.0	0 (0)	0 (0)	8	1.0 ± 1.9	12 (30)	4 (10)	7	0.3 ± 1.2	3 (7.5)	1 (2.5)
Headache	1	0.1 ± 0.2	2 (5)	0 (0)	6	0.4 ± 1.2	6 (15)	1 (2.5)	5	0.3 ± 1.1	4 (10)	2 (5)
Muscle Cramps	0	0.0 ± 0.0	0 (0)	0 (0)	9	2.4 ± 2.8	23 (57.5)	12 (30)	7	0.9 ± 1.5	15 (37.5)	2 (5)
Urge to Urinate	4	0.2 ± 0.8	4 (10)	1 (2.5)	7	1.3 ± 2.0	15 (37.5)	6 (15)	3	0.3 ± 0.8	4 (10)	1 (2.5)
Thirsty	4	0.7 ± 1.1	14 (35)	1 (2.5)	8	2.7 ± 2.7	25 (62.5)	15 (37.5)	7	1.2 ± 1.6	20 (50)	4 (10)
Fever	0	0.0 ± 0.0	0 (0)	0 (0)	2	0.1 ± 0.3	1 (2.5)	2 (5)	1	0.0 ± 0.2	1 (2.5)	1 (2.5)
Swollen Hands	0	0.0 ± 0.0	0 (0)	0 (0)	3	0.2 ± 0.7	5 (12.5)	2 (5)	2	0.1 ± 0.4	3 (7.5)	0 (0)
Swollen Feet	0	0.0 ± 0.0	0 (0)	0 (0)	5	0.5 ± 1.2	6 (15)	2 (5)	5	0.4 ± 1.1	6 (15)	2 (5)
Tired/Fatigued	2	0.1 ± 0.3	2 (5)	0 (0)	9	4.2 ± 3.1	30 (75)	22 (55)	7	2.6 ± 2.2	31 (77.5)	15 (37.5)
Muscle Soreness/Weakness	4	0.2 ± 0.7	3 (7.5)	1 (2.5)	9	4.0 ± 3.1	30 (75)	21 (52.5)	8	4.1 ± 2.3	38 (95)	23 (57.5)
Tingling in Arms	0	0.0 ± 0.0	0 (0)	0 (0)	2	0.1 ± 0.4	2 (5)	1 (2.5)	0	0.0 ± 0.0	0 (0)	0 (0)
Tingling in Legs	0	0.0 ± 0.0	0 (0)	0 (0)	2	0.1 ± 0.5	2 (5)	2 (5)	1	0.0 ± 0.2	1 (2.5)	0 (0)

Abbreviations: M = mean; SD = standard deviation; L = left; R = right.

Notes: Overall incidence was determined by the absolute number of incidences of a symptom occurring at any time point and reported as a percentage. Scores > 4 are considered "serious". Percent serious calculated as the number of sessions with a score > 4 out of the total number of sessions at that time point. Max is the maximum score reported for that symptom.
