# Supplementary material for: Gastrointestinal cell injury and perceived symptoms after running the Boston Marathon
Source: Front Physiol. 2023 Oct 16;14:1268306. doi: 10.3389/fphys.2023.1268306 (PMC10615131; doi:10.3389/fphys.2023.1268306)
Supplement: Supplementary file 3 [file Table1.pdf]

Supplemental Table 1. GI Responses and risk factors present for each subject ranked in order starting with greatest %ICI decrease from post- to 24-hours post-race.

	I-FABP (pg/ml)			GI Symptoms			Risk Factors Present										
	%ICI	Post	24 hrs	Pre	Post	24 hrs	Alcohol Mon.	EHI	Rx	OTC	Supp.	Ill	NSAIDs Post	Sleep Post	Water during	Travel	# Risk Factors
1	-90.0	4300.1	429.6		✓	✓						✓				✓	2
2	-88.5	9183.8	1058.1		✓	✓	✓			✓	✓					✓	4
3	-86.7	14086.6	1878.1	✓	✓	✓	✓		✓		✓		✓				4
4	-84.4	4070.8	635.1	✓	✓	✓							✓			✓	2
5	-84.4	4765.4	743.7		✓	✓					✓			✓		✓	3
6	-77.6	3714.4	832.6		✓	✓										✓	1
7	-69.5	6209.4	1895.6	✓	✓	✓	✓				✓		✓			✓	4
8	-66.7	6548.4	2182.8	✓	✓	✓	✓		✓	✓	✓			✓	✓	✓	7
9	-66.6	3122.6	1041.9		✓	✓	✓				✓					✓	3
10	-62.8	3053.8	1136.9	✓	✓	✓	✓				✓					✓	3
11	-62.7	3924.7	1465.3	✓		✓	✓	✓			✓						3
12	-59.1	2068.1	845.5		✓	✓	✓									✓	2
13	-57.9	5868.6	2468.8				✓		✓					✓		✓	4
14	-51.6	2057.0	995.4	✓	✓	✓		✓	✓	✓	✓		✓			✓	6
15	-51.3	1669.0	813.7	✓	✓	✓	✓		✓		✓				✓	✓	5
16	-50.0	2484.6	1243.5		✓	✓	✓				✓					✓	3
17	-49.9	9347.2	4680.3	✓	✓	✓			✓		✓						2
18	-45.7	2123.6	1154.0		✓	✓	✓				✓					✓	3
19	-44.4	2250.3	1252.1		✓	✓					✓					✓	2
20	-44.3	2698.8	1503.6		✓	✓	✓				✓					✓	3
21	-43.8	3825.9	2152.2	✓	✓	✓		✓	✓		✓	✓		✓		✓	6
22	-40.2	1400.9	837.3		✓	✓					✓					✓	2
23	-39.1	2853.9	1739.4		✓	✓	✓									✓	2
24	-36.9	2227.0	1405.1		✓	✓	✓				✓	✓				✓	4
25	-36.1	2394.4	1529.5	✓	✓	✓							✓			✓	2
26	-31.9	1885.0	1284.5		✓	✓	✓				✓	✓				✓	4

Supplemental Table 1 Continued

	I-FABP (pg/ml)			GI Symptoms			Risk Factors Present										
	%ICI	Post	24 hrs	Pre	Post	24 hrs	Alcohol Mon.	EHI	Rx	OTC	Supp.	Ill	NSAIDs Post	Sleep Post	Water during	Travel	# Risk Factors
27	-27.4	1898.9	1378.7	✓	✓	✓					✓					✓	2
28	-25.6	3429.7	2552.5	✓	✓	✓	✓			✓	✓					✓	4
29	-20.6	1390.0	1103.9	✓	✓	✓	✓	✓	✓		✓	✓		✓		✓	7
30	-11.6	2092.0	1849.1		✓	✓	✓	✓									2
31	-7.1	1664.8	1547.1	✓	✓	✓	✓	✓	✓		✓					✓	5
32	0.1	2505.3	2507.4		✓	✓	✓									✓	2
33	6.4	1251.2	1331.5	✓	✓	✓	✓				✓						2
34	10.4	1169.0	1291.0	✓	✓	✓		✓			✓			✓		✓	4
35	12.4	1192.6	1340.5	✓	✓	✓	✓						✓			✓	3
36	16.7	1782.2	2080.5	✓	✓	✓				✓	✓		✓	✓		✓	5
37	17.0	3175.3	3716.0	✓	✓	✓	✓				✓			✓			3
38	58.6	964.1	1529.2		✓	✓	✓	✓	✓		✓		✓			✓	6
39	68.5	2807.3	4729.5		✓	✓	✓		✓	✓	✓			✓		✓	6
40	71.3	1243.6	2129.9	✓	✓	✓	✓				✓					✓	3

Abbreviations: %ICI = percent change from post- to 24-hours post-race gastrointestinal permeability; I-FABP = intestinal fatty acid binding protein; GI = gastrointestinal; h = hour; Mon. = Monday; EHI = exertional heat illness; Rx = prescription medication; OTC = over-the-counter medication; NSAIDs = non-steroidal anti-inflammatory drugs; Supp. = supplement

Notes: A "✓" indicates the presence of GI symptoms reported at each time point OR if the participant reported a risk factor that could potentially impact their recovery from post- to 24 h post-race. Previous history of EHI included previous history of any exertional heat illness, exertional heat exhaustion, and exertional heat stroke; prescription and OTC medications and supplement use included reports of any medication or supplements; illness refers to reported current illness <5 days before the race; sleep since post-race was ✓ if they reported sleeping <5 hours or rated their sleep as "below average"; if they did not consume water during the race; and if they traveled to the race. Total number of risk factors present by subject is in the “# Risk Factors” column.
