# Supplementary material for: Gastrointestinal cell injury and perceived symptoms after running the Boston Marathon
Source: Front Physiol. 2023 Oct 16;14:1268306. doi: 10.3389/fphys.2023.1268306 (PMC10615131; doi:10.3389/fphys.2023.1268306)
Supplement: Supplementary file 4 [file Table2.pdf]

Supplemental Table 2. Average macronutrients overall per day and by sex

	Calories (kcal)		Protein (g)		Carbohydrates (g)		Fats (g)	
	mean ± SD	min - max	mean ± SD	min - max	mean ± SD	min - max	mean ± SD	min - max
**Sunday**								
Overall	2372.3 ± 963.2	776.7 - 5861.5	90.0 ± 35.2	30.2 - 174.4	330.3 ± 178.4	108.4 - 1129.7	75.3 ± 36.0	13.3 - 151.5
Male	2576.7 ± 1183.5*	776.7 - 5861.5	94.8 ± 39.0	30.2 - 174.4	385.4 ± 232.0†	133.5 - 1129.7	74.7 ± 36.6	13.3 - 151.5
Female	2187.3 ± 687.7	1097.4 - 3415.6	85.6 ± 31.8	42.9 - 165.7	280.4 ± 90.1	108.4 - 457.0	75.8 ± 36.3	19.5 - 151.2
**Monday**								
Overall	2938.6 ± 1098.4	1038.8 - 5763.9	82.9 ± 35.5	16.0 - 179.4	410.9 ± 176.7	159.7 - 787.3	86.7 ± 46.4	20.6 - 272.7
Male	3341.8 ± 1178.1	1038.8 - 5763.9	93.2 ± 32.9	26.7 - 179.4	489.6 ± 182.0	186.8 - 787.3	94.5 ± 58.5	20.6 - 272.7
Female	2573.9 ± 899.5	1066.9 - 4065.3	73.5 ± 35.9	16.0 - 163.6	339.8 ± 141.1	159.7 - 647.0	79.6 ± 31.9	21.9 - 156.1
**Tuesday**								
Overall	2313.9 ± 860.5	1079.3 - 3898.7	90.5 ± 36.0	36.0 - 157.1	268.5 ± 117.0	93.1 - 561.3	86.4 ± 35.6	32 - 170.8
Male	2551.3 ± 909.9	1079.3 - 3898.7	99.7 ± 39.2	36.0 - 157.1	299.4 ± 134.0	109.1 - 561.3	97.6 ± 36.6	32.0 - 156.7
Female	2135.8 ± 798.2	1206.6 - 3523.2	83.6 ± 32.8	37.3 - 151.8	245.4 ± 99.7	93.1 - 399.2	78.0 ± 33.3	36.2 - 170.8

Abbreviations: kcal = kilocalories; g = grams; SD = standard deviation; min = minimum; max = maximum.
Notes: Males consumed significantly more *calories ($t(38) = 2.330$, $p = .025$, $d = .699$) and †carbohydrates ($t(38) = 2.925$, $p = .006$, $d = .848$) than females.
