# Supplementary material for: Gastrointestinal cell injury and perceived symptoms after running the Boston Marathon
Source: Front Physiol. 2023 Oct 16;14:1268306. doi: 10.3389/fphys.2023.1268306 (PMC10615131; doi:10.3389/fphys.2023.1268306)
Supplement: Supplementary file 5 [file Table3.pdf]

Supplemental Table 3. Absolute I-FABP and %ICI responses across demographics and risk factors

		%ICI Change		I-FABP Post-Race		I-FABP 24-h Post-Race	
	n	Mean ± SD	*p*	Mean ± SD	*p*	Mean ± SD	*p*
**Screening and baseline survey**							
Males	19	-34.8 ± 39.1	.886	3612.8 ± 2119.8	.575	1944.4 ± 1255.4	.085
Females	21	-32.9 ± 45.3		3145.6 ± 3061.2		1397.5 ± 441.3	
Traveled to race	34	-31.2 ± 41.3	.874	2965.4 ± 1808.0	.250	1510.9 ±796.1	.146
Did not travel	6	-34.3 ± 42.3		5646.1 ± 5019.1		2486.7 ± 1376.8	
History of EHI	8	-16.0 ± 38.9	.181	2135.9 ± 1143.3	.023*	1491.6 ± 379.1	.372
No history of EHI	32	-38.3 ± 41.6		3675.4 ± 2817.9		1698.8 ± 1046.8	
No illness past 5 days	35	-32.3 ± 43.4	.406	3459.2 ± 2774.3	.341	1711.9 ± 983.4	.216
Illness past 5 days	5	-44.6 ± 26.7		2725.6 ± 1267.8		1275.0 ± 618.6	
Not taking Rx med	26	-36.4 ± 38.2	.620	2695.2 ± 1227.0	.095	1404.6 ± 662.1	.056
Taking Rx med	14	-29.0 ± 48.5		4616.0 ± 3915.1		2126.6 ± 1226.2	
Not taking OTC med	34	-35.5 ± 38.9	.674	3202.7 ± 2588.2	.422	1549.8 ± 841.4	.261
Taking OTC med	6	-24.5 ± 58.2		4301.4 ± 2941.5		2266.5 ± 1361.3	
Not taking supplement	30	-30.3 ± 43.5	.323	3454.7 ± 2950.8	.618	1737.1 ± 1010.7	.294
Taking supplement	10	-44.3 ± 35.8		3106.0 ± 1376.6		1417.7 ± 734.8	
**Pre-race survey**							
No NSAIDs night before race	35	-33.0 ± 43.8	.590	3379.7 ± 2734.7	.926	1653.7 ± 1005.9	.917
NSAIDs night before race	5	-39.9 ± 22.2		3281.7 ± 2010.5		1682.0 ± 458.4	
Sleep before race <5 or "below avg"	10	-34.8 ± 55.8	.944	4116.5 ± 2412.4	.290	1878.6 ± 1183.8	.482
Sleep before race >5 or "avg+"	30	-33.5 ± 36.9		3117.8 ± 2695.0		1583.5 ± 870.7	
**Post-race survey**							
Reported medical event during race	14	-30.0 ± 37.0	.661	2875.5 ± 2119.4	.350	1676.8 ± 1059.8	.929
No medical event during race	26	-35.9 ± 44.5		3632.4 ± 2876.6		1646.8 ± 908.6	
Believed hydrated	38	-33.6 ± 41.3	.955	3406.8 ± 2676.7	.684	1693.8 ± 958.9	.223
Did not believe hydrated	2	-37.0 ± 67.1		2619.9 ± 2051.9		963.0 ± 463.8	
Drank water during race	38	-32.5 ± 42.3	.079	3328.5 ± 2637.7	.803	1665.6 ± 962.2	.848
Did not drink water during race	2	-59.0 ± 10.9		4108.7 ± 3450.3		1498.2 ± 968.1	
Ate food during race	13	-31.3 ± 44.0	.804	3361.3 ± 2323.2	.993	1704.5 ± 1040.1	.839
Did not eat food during race	27	-35.0 ± 41.2		3370.5 ± 3296.4		1634.5 ± 924.6	

Supplemental Table 3 Continued

		%ICI Change		I-FABP Post-Race		I-FABP 24-h Post-Race	
	n	Mean ± SD	*p*	Mean ± SD	*p*	Mean ± SD	*p*
**24-h survey**							
No NSAIDs since the race	32	-34.8 ± 39.1	.821	3185.7 ± 2049.9	.585	1700.2 ± 1036.3	.402
NSAIDs since the race	8	-30.1 ± 53.5		4094.6 ± 4390.3		1485.5 ± 487.7	
Sleep after the race <5 or "below avg"	9	-17.9 ± 49.7	.276	3481.3 ± 1937.4	.860	2274.2 ± 1269.1	.105
Sleep after the race >5 or "avg+"	31	-36.5 ± 38.7		3334.4 ± 2830.2		1478.1 ± 773.4	
**Usg**							
Pre-race hydrated	38	-33.7 ± 40.9	.965	3384.7 ± 2686.7	.832	1678.4 ± 952.2	.698
Hypohydrated	2	-36.6 ± 75.5		3041.2 ± 1780.4		1255.1 ± 1167.3	
Post-race hydrated	33	-34.8 ± 42.5	.816	3396.0 ± 2800.6	.840	1640.1 ± 994.9	.917
Hypohydrated	6	-30.1 ± 44.0		3200.4 ± 1964.5		1602.7 ± 741.4	
24-h hydrated	26	-31.6 ± 41.0	.662	3181.8 ± 2872.2	.517	1492.0 ± 526.3	.249
Hypohydrated	14	-37.9 ± 44.0		3712.3 ± 2178.1		1964.2 ± 1422.2	
**Alcohol consumption**							
Sunday Yes	9	-29.4 ± 46.6	.744	2810.6 ± 1533.7	.332	1573.7 ± 657.6	.710
No	31	-35.1 ± 40.8		3529.2 ± 2875.5		1681.5 ± 1028.1	
Monday Yes	27	-27.8 ± 44.3	.151	3397.2 ± 2867.4	.912	1757.5 ± 873.7	.387
No	13	-46.4 ± 33.6		3305.9 ± 2172.6		1449.1 ± 1101.9	
**Sum risk factors**							
1 to 2	13	-42.6 ± 30.3		3088.3 ± 2106.5		1526.8 ± 1103.5	
3 to 4	18	-39.0 ± 42.5	.097	3991.3 ± 3243.7	.750	1628.3 ± 718.6	.382
5+	9	-10.8 ± 49.3		2523.2 ± 1729.5		1903.8 ± 1176.7	

Abbreviations: GI = gastrointestinal; I-FABP = intestinal fatty acid binding protein; %ICI = percent change in intestinal cell injury from post-race to 24-hours post-race; SD = standard deviation; 24-h = 24-hour post-race; Rx = prescription; OTC = over-the-counter; NSAIDs = non-steroidal anti-inflammatory drugs; avg = average; Usg = urine specific gravity.

Notes: Risk factor results are from independent samples t-tests and sum risk factors is based on one-way ANOVA.
